# Contexts motivating protective behaviours related to *Aedes*-borne infectious diseases in Curaçao

**DOI:** 10.1186/s12889-023-16624-5

**Published:** 2023-09-05

**Authors:** Vaitiare Mulderij-Jansen, Izzy Gerstenbluth, Ashley Duits, Adriana Tami, Ajay Bailey

**Affiliations:** 1 Department of Medical Microbiology and Infection Prevention, University of Groningen, University Medical Center Groningen, Groningen, The Netherlands; 2 International Development Studies, Department of Human Geography and Spatial Planning, Faculty of Geosciences, Utrecht University, Utrecht, Netherlands; 3 Department of Epidemiology, Curaçao Biomedical & Health Research Institute, Willemstad, Curaçao; 4 Institute for Medical Education, University Medical Center Groningen, Groningen, The Netherlands; 5 Department of Immunology, Curaçao Biomedical & Health Research Institute, Willemstad, Curaçao; 6 Red Cross Blood Bank Foundation, Willemstad, Curaçao

**Keywords:** Caribbean Region, Dengue, Zika virus, Chikungunya virus, Mosquito control, Health belief model, Environment and public health

## Abstract

**Background:**

*Aedes aegypti*, the vector of arboviral diseases such as dengue and Zika virus infections, is difficult to control. Effective interventions must be practicable, comprehensive, and sustained. There is evidence that community participation can enhance mosquito control. Therefore, countries are encouraged to develop and integrate community-based approaches to mosquito control to mitigate *Aedes*-borne infectious diseases (ABIDs). Health professionals must understand the contexts motivating individuals’ behaviour to improve community participation and promote behavioural change. Therefore, this study aimed to determine how contexts shaped individuals’ protective behaviours related to ABIDs in Curaçao.

**Methods:**

From April 2019 to September 2020, a multi-method qualitative study applying seven (*n* = 54) focus group discussions and twenty-five in-depth interviews with locals was performed in Curaҫao. The study was designed based on the Health Belief Model (HBM). Two cycles of inductive and deductive coding were employed, and Nvivo software was used to manage and analyse the data.

**Results:**

In this study, low media coverage (external cue to action) and limited experience with the symptoms of ABIDs (internal cue to action) were linked with a low perceived susceptibility and severity of ABIDs (low perceived threat). The low perceived threat was linked with reduced health-seeking behaviour (HSB) to prevent and control ABIDs. We also found that the perceived barriers outweigh the perceived benefits of ABID prevention and control interventions, obstructing HSB. On the one hand, insufficient knowledge reduced self-efficacy but contrary to expected, having good knowledge did not promote HSB. Lastly, we found that our participants believe that they are responsible for preventing ABIDs (internal locus of control) but at the same time indicated that their success depends on the efforts of the community and the health system (external locus of control).

**Conclusions:**

This study used the HBM to explain individual changes in HSB concerning ABIDs prevention and control in Curaçao. We can conclude that the perceived threat (perceived susceptibility and severity) and perceived barriers played an essential role in changing HSB. Health professionals must consider these two concepts' implications when designing a bottom-up approach for ABIDs control; otherwise, community participation will remain minimal.

**Supplementary Information:**

The online version contains supplementary material available at 10.1186/s12889-023-16624-5.

## Introduction

*Aedes (Ae.) aegypti* and *Aedes* *albopictus* mosquitoes have been implicated as potential vectors of dengue, chikungunya and Zika viruses [1, 2]. In the Latin American region, the primary vector of these viruses is the *Ae. aegypti* mosquito [1]. *Aedes*-borne infectious diseases (ABIDs) are expanding their spatial range, gradually emerging in previously unaffected areas and re-emerging in regions where these diseases have been eradicated [3]. The prevention and control of the ABIDs mentioned above rely mainly on mosquito control because no antiviral treatment nor vaccines suitable for large-scale use are available [4]. There is a licensed dengue vaccine (Dengvaxia vaccine); however, it is not widely used due to safety concerns [5]. The World health Organization (WHO) recommends Dengvaxia for persons aged 9–45 years with confirmed previous dengue virus infection. Individuals not previously infected with dengue virus who receive the vaccine might be at risk of developing severe dengue if infected with the mentioned virus after being vaccinated because of the antibody-dependent enhancement phenomenon [6, 7]. No approved vaccines are available for Zika and chikungunya [8, 9]. Trials of Zika and chikungunya virus vaccines are ongoing. Some temporary successes (e.g., reduction of entomological indices, mosquito breeding sites, and cases of ABIDs) have been achieved; however, these have not persisted, partly because of the top-down approach, which was often vertically structured, without community involvement, and costly to be sustainable [10–12].

In the 1990s, the public health approach combining active surveillance, emergency response, case management, and community-based mosquito control became the basis for the World Health Organization global strategy for preventing and controlling ABIDs [13]. Such an integrated approach has been implemented in different countries, and beneficial results have been reported [14, 15]. Efforts have been made to implement integrated mosquito management in Curaҫao. Nevertheless, successful program implementation has remained a challenge. Insufficiently qualified workforce, resources and engagement from the health authorities and communities have obstructed the implementation of mosquito control interventions [16].

Based on the failures of the top-down mosquito control approach, countries are encouraged to emphasise the bottom-up approach. The bottom-up approach refers to an integrated community-based approach to mosquito control by environmental management, emphasising health education and community ownership [17]. The main aim of this approach is to convince people to take ownership of household and neighbourhood mosquito control. To improve community participation and promote behavioural change, health professionals need to understand the community’s behaviour. Therefore, this study was designed to determine perceptions, beliefs and attitudes motivating individuals’ protective behaviours related to ABIDs in Curaçao. This study is part of a larger research project, the ARBOCARIB project, where health system preparedness and performance, risk communication, social amplification of risk and individual protective behaviour regarding ABIDs have been studied [16, 18, 19].

Data from our previous research has highlighted how the macro-level (e.g., the performance of the health system concerning mosquito control and risk communication) and meso-level (e.g., social influence, cultural schemas) shaped individual protective behaviour concerning ABIDs in Curaçao [18, 19]. Published data from our previous research support the literature suggesting that people’s perceptions and actions are shaped by their environmental and socio-cultural context [20]. Besides the macro and the meso-level, the micro-level (individual level) also needs to be explored. Therefore, in this current study, the Health Belief Model (HBM) will be applied to understand better the intricate processes underlying individuals’ decision-making and health-seeking behaviour (HSB). We will explore the role of the following key concepts of the HBM (i) perceived susceptibility, (ii) perceived severity, (iii) perceived barriers, (iv) perceived benefits, and (v) self-efficacy on HSB [21]. HSB in this study refers to any action or inaction undertaken by individuals to prevent or control ABIDs spread. The influence of cues to action (e.g., risk information) on the perceived threat (perceived susceptibility and severity) and self-efficacy will also be studied. Health professionals can use our findings to work towards more bottom-up *Aedes* control interventions.

## Methods

**Fig. 1:**
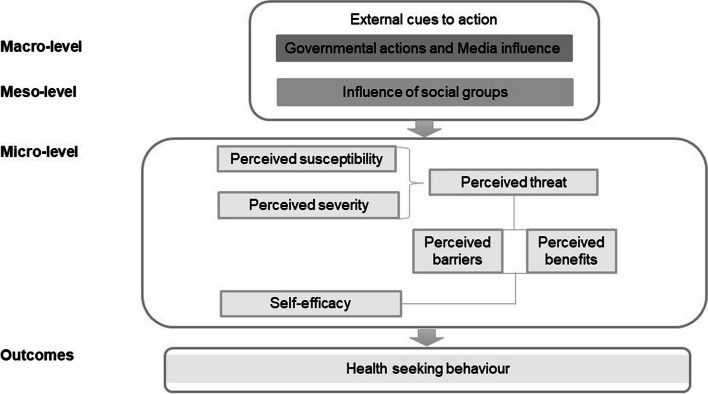
A conceptual framework for understanding individuals’ decision-making process and health-seeking behaviour regarding ABIDs

From April 2019 to September 2020, a multi-method qualitative study using focus group discussions (FGDs) and in-depth interviews (IDIs) with locals was performed in Curaҫao. The study was designed based on the theoretical framework of the HBM [21]. In previous research, we have used some concepts of the HBM (perceived susceptibility, perceived severity and cues to action) to study the perceived threat of ABIDs [18, 19]. However, this study goes a step forward by using the complete HBM to explain the underlying motivations for multifaceted and complex individuals’ health-seeking decisions. The concept of external cues to action represents the influences of the macro and the meso-level on the perceived threat (perceived susceptibility and perceived severity) and self-efficacy. The study’s conceptual framework was applied during data collection, analysis, and interpretation of results (Fig. 1).

### Study population

This study was conducted on the Dutch Caribbean Island called Curaçao, located ± 65 km north of the Venezuelan coast. The island has a surface area of 444 km^2^ and approximately 158,665 inhabitants on January 1st, 2019 [22]. There are different ethnic backgrounds, with an Afro-Caribbean majority and minorities such as Dutch, French, Latin American, South- and East- Asian, Portuguese and Levantine people [23]. The official languages of Curaçao are Papiamentu, Dutch and English. However, Spanish is also widely spoken.

Study participants were recruited using the snowball recruitment technique, key informants, and via community centres. The participants recruited met the following criteria: they live and had lived on the island during at least one of the outbreaks (the last dengue outbreak occurred in 2010, the chikungunya outbreak in 2014–2015 and the Zika outbreak in 2016), reported having experienced an ABID or know someone who experienced an ABID and had been bitten by mosquitoes. Men and women aged 17–86 years, with low, middle, and high socioeconomic status (SES), different ethnic backgrounds (including Afro-Caribbean, Dutch, Latin American, and Portuguese), and religious beliefs participated in our study. The participant's addresses are scattered throughout the island. The participants were encouraged to share information about their family, including family structure and living environment. Based on the data collected, people living in affluent and poor neighbourhoods participated in our study. We considered all these aspects when sampling our study population, which is why we believe that our study population represents the whole community.

We collected data by conducting FGDs and IDIs on different individuals. Seven population groups of Curaҫao were selected to participate in FGDs (*n* = 54). The following groups were included: (i) local youth (high school students aged 15–20 years), (ii) individuals from the neighbourhood of Seru Papaya, (iii) Adventists, (iv) Jehovah’s witnesses, (v) Protestants, (vi) Catholics and, (vii) employees of an insurance company. In total, 25 newly recruited individuals with different SES were interviewed (participants of the in-depth interviews) (see Tables 1 and 2). The participant’s reported education and occupation were used as a proxy to determine their SES. In this study, an individual with a low SES reported finishing primary school and was unemployed at the moment of data collection. An individual with a middle SES reported having a paid job or receiving a retirement fee and finishing secondary or intermediate vocational education. Individuals with a high SES reported having a paid job and finishing higher vocational education or university.

**Table 1:** Characteristics of the study participants of the FGDs with the community

FGDs (*n* = 7)	(n) participants	Gender^**a**^ F:M	Age range	SES^**b**^
	(*n* = 54)			
Local youth^c^	8	2:6	17–20	High
Seru Papaya	12	12:0	42–73	Low-middle
Adventists	6	3:3	35–69	Middle-high
Jehovah witnesses	4	4:0	39–62	Low-middle
Protestants	8	4:4	33–67	Middle-high
Catholics	11	11:0	55–86	Low-middle-high
Employees of an insurance company	5	3:2	37–62	Middle-high

^a^*F* Female, *M* Male

^b^*SES* socioeconomic status

^c^The parents of the students have a high SES

**Table 2:** Characteristics of the study participants of the IDIs

IDIs (*n* = 25)	SES^b^	Gender^a^	Age
01	Middle	F	35
02	High	F	59
03	High	F	29
04	Middle	F	28
05	Middle	M	30
06	Middle	F	49
07	Middle	F	57
08	High	F	54
09	High	F	67
10	Low	F	63
11	Middle	F	81
12	Middle	F	46
13	Low	F	40
14	Middle	F	46
15	Middle	M	40
16	Middle	F	58
17	High	M	59
18	Middle	M	52
19	Middle	M	55
20	Low	F	55
21	Middle	F	46
22	Middle	M	35
23	High	F	64
24	Middle	F	22
25	Middle	F	25

^a^*F* Female and *M* male

^b^*SES* socioeconomic status

### Data collection

FGDs were conducted first to gain an in-depth understanding of social issues regarding community participation in mosquito control. Seven FGDs with different social groups were performed. Twenty-five IDIs were conducted in the second phase of data collection to verify and support data drawn from the FGDs. The topic guides for the FGDs and IDIs were semi-structured to cover each concept of the conceptual framework of this study (See Additional files 1 and 2: Text S1-2 Topic guides FGD and IDIs). The topic guides were piloted and adjusted before the data collection. The FGDs and IDIs were conducted in Papiamentu or Dutch, recorded, and transcribed.

### Data analysis

The data collected from FGDs and IDIs with community members were analysed inductively (codes raised by the study participants themselves) and deductively (codes derived from literature and existing theories) (Additional file 3: Table S1. Coding list). Two cycles of inductive and deductive coding were employed in the data analyses of the FGDs. In the first cycle, the data was assigned to codes, and these codes were assigned to 12 categories, which were studied in the second cycle of data analysis. We created the following categories by combining inductive and deductive codes: (i) knowledge about ABIDs, (ii) perceived susceptibility, (iii) perceived severity, (iv) perceived barriers, (v) perceived benefits, (vi) self-efficacy, (vii) trust, (viii) internal locus of control, (ix) external locus of control, (x) cues to action, (xi) HSB, and (xii) recommendations. The same method was used to analyse the IDIs with locals. Similar codes and categories were used to compare the data from the FGDs and IDIs. This data analysis method facilitated the data interpretation to elicit meaning, compare data, gain comprehended understanding and develop empirical knowledge. The data was analysed using Nvivo software (version 12 Pro). All quotes used in this article were transliterated to keep the context intact.

## Results

Seven FGDs (*n* = 54) and 25 IDIs were conducted. Thirty-nine participants of the FGDs (72%) and nineteen interviewees (76%) were female. About two-thirds (64%) of the interviewees had a middle SES, and the mean age of the interviewees was 47.8 years. The characteristics of the interviewed community members are presented in Tables 1 and 2.

Over 75 codes were gathered into 12 categories organised into five themes. The themes refer to the concepts that influence decision-making processes and behaviour concerning the prevention and control of ABIDs. The following five concepts are discussed in this article: (i) perceived threat, (ii) perceived barriers, (iii) self-efficacy and (iv) internal and external locus of control, and (v) HSB.

### Perceived threat

People associated high media coverage with high disease susceptibility and severity. Most participants from the FGDs and IDIs indicated that ABIDs were/are not a threat to their lives because limited attention from the government/health system and media channels was given to these diseases before, during and after the last three epidemics (dengue 2010, chikungunya 2014–2015, and Zika 2016). The following quotes portray this issue.

‘Interviewer: Okay, do you feel at risk for one of these diseases?

Female G: Hmm, no, no, I do not feel that. It is weird actually because the mosquitoes are still here. We still see the mosquitoes, but we are not paranoid about them anymore.

*Interviewer: Okay.*

Female G: But you do not know when there is another outbreak.

Interviewer: And can you explain why not? Because you said, the mosquitoes are still here.

Female G: Because you do not hear anything within the community or of infected people. Maybe there are people with the disease, but no information is given (information coming from the health system). These diseases are not relevant anymore, and you do not pay attention to them anymore. You go back to your everyday life.

P08- Female, inspectress (IDI).

‘Interviewer: Okay, let us continue with the first topic. There are three topics. The first topic is risk perception, thus the feeling of being at risk of the disease. For example, do you feel at risk of these diseases?

*Female T: Not now.*

*Interviewer: No? Why not?*

Female T: Because nobody talks about them.

P25- Female, Social worker (IDI).

When discussing the media coverage of disease in general, some participants of the IDIs indicated that COVID-19 is more dangerous than ABIDs because the health system and the media channels shared more information about COVID-19 and its control measures than they did during the ABID epidemics. The following quote portrays this issue:

‘Interviewer: Why do they pay more attention to COVID-19? They use all types of media channels nowadays. Why do you think this did not happen during those epidemics?

Female T: Hmm. well, what I already said, it is the seriousness/ severity of the.

*Interviewer: The disease?*

*Female T: The disease, yes.*

*Interviewer: Aha.*

Female T: Maybe the symptoms of those diseases are mild, and you recover faster.

*Interviewer: Aha.’*

P25- Female, Social worker (IDI).

Women had a higher perceived threat of ABIDs than men, especially for Zika virus infection, because of the possible pregnancy complications in the newborn (microcephaly) that were mentioned in the media. Most of the study participants of the IDIs and FGDs, including high school students who participated in an FGD, reported that women had a higher perceived threat of Zika than men or the younger generation.

‘Female 3: The worry remains. I was worried about being bitten. What if I was bitten, and what will happen to the baby. I wanted to know if the baby was okay when the baby was born. So the worry remains.

Moderator: Can I ask something?

*Female 3: Oh yeah, sure.*

Moderator: So, were women more worried than men during the Zika epidemic? Because you (women) can be pregnant?

*Female 3: Yes.*

Moderator: Do you all believe so?

Female 2: Yes, I remember that they recommended people (women) not to get pregnant that year.

*Moderator: Okay.’*

-FGD with Jehovah's Witnesses.

Besides women, people who acquired ABIDs or experienced how a close relative dealt with these diseases also reported being afraid and motivated to perform ABIDs prevention and control activities. In other words, the disease was not considered a threat or a priority when its implications did not directly affect that person (internal cue to action) or a close relative/people they knew. In this study, internal (being sick) and external cues to action (media coverage/ information coming from the community) triggered the decision to perform mosquito control measures. Thus, a lack of internal and external cues to action was linked with a low perceived threat and motivation to conduct prevention and control activities.

### Perceived barriers

Different barriers that negatively influenced people’s decision-making and HSB were mentioned during the IDIs and FGDs. One frequently mentioned barrier was the limited governmental involvement in mosquito control. Many participants indicated that the government had a passive attitude toward mosquito control in the last three ABID epidemics. The mentioned perception is portrayed by the quote below.

‘Male L: Honestly, this is more a gut feeling; they (the government) fogged (spray insecticide) not to get rid of the problem, but for propaganda, the community wanted something to happen. So fog, see? We did something.

Interviewer: A false sense of security?

Male L: Yeah, that was the feeling I had, especially in the Zika epidemic. I did not see many activities during the period of chikungunya.’

*P19, Male, teacher (IDI).*

Mosquito control is challenging, but the lack of governmental and community involvement in mosquito control makes it harder to prevent and control outbreaks of ABIDs. There is a lack of collaboration between the government and the community. Most interviewees indicated that the vector control department did not act proactively, and some even thought that the department was not operating anymore. Furthermore, some people were unaware of the health system’s services (e.g., free distribution of Abate and Gambusia fish). Participants of the FGDs further elaborated on this topic and indicated that the government’s passive attitude did not motivate them to continue with needed interventions to control mosquitoes.

The community’s passive attitude towards mosquito control also negatively influenced individuals’ preventive behaviour. Some participants of both FGDs and IDIs indicated that many people removed mosquito breeding sites. However, a group of people did not perform the mentioned activity. The negligence of others led to more mosquitoes. Therefore, the efforts to reduce mosquito breeding sites were considered less effective. The following quote portrays this issue:

‘Male 3: We are a community; it is sad to say so, but we do not like keeping our environment clean.

Moderator: We do not like to maintain the environment clean?

*Male 3: laughs*

Male 3: Maybe it sounds like a joke, but I am honest.

*Female 1: Hmm, that is true.*

Male 3: We create conditions that can increase the chance for issues. Although they often say remove items that collect water, I cover and turn items that can collect the water; you can live close to me, and you are not doing these things.

Moderator: So the situation remains the same (no effect)?

Male 3: Yes, so there are many mosquito breeding sites.

*Female 1: Yes.*

*-FGD with Adventists.*

The perception that mosquito control is challenging has reduced the motivation to act. The issue with waste management in Curaçao also has amplified the feeling that it is impossible to control the mosquitoes. Waste scattered throughout the island is considered potential mosquito breeding sites. Some participants indicated that they could clean up their gardens, but if there is waste in their neighbourhoods, all their efforts to reduce mosquito breeding sites are useless.

Furthermore, limited knowledge about control strategies was another barrier to preventing and controlling mosquitoes. Some people believe that the usage of vitamins is enough to prevent ABIDs. Besides, poverty and insufficient access to the health system to receive information and support with mosquito control strategies also influenced the preventive behaviour of some study participants negatively. This issue became evident during a FGDs with people living in a low-middle social-economic neighbourhood.

‘Moderator: When you live in poverty, prevention is not your priority? That is what you are saying?

*Female 9: No.*

Moderator: Your priority is to survive?

Female 9: Yes, to survive. Although we want to think about prevention, it remains at that stage, thinking because it is not something you can do. Many people that live here are aware of this issue. Many want to get the Abate (larvicide offered by the Health System) or go to a presentation about mosquito control. However, if the option is not close to you, you cannot reach it.’

-FGD, Community centre Seru Papaya.

The participants of the FGD conducted at the Community centre Seru Papaya also indicated that they did not have enough financial resources to maintain their houses free of mosquito breeding sites during the epidemics. For example, some reported having damaged cesspools that could also be potential mosquito breeding sites. Lastly, some participants of the IDIs indicated that they did not use repellents due to the bad smell and the product build-up on their bodies.

### Self-efficacy

Due to a lower perception of benefits, the willingness of people to take action was also reduced. When the participants were asked about their confidence in their ability to take action to control mosquitoes, the majority of the participants had good knowledge about the control measures. They felt confident that their actions were suitable to control the mosquito population. However, a few participants indicated that they did not know how to prevent ABIDs, due to a lack of information.

‘Interviewer: Okay, thus no action or consequences affected you? Hmm, currently, are you doing something to control mosquitoes?

Female S: There is not so much you can do to control the mosquitoes. At least I do not know what I can do. If there is something, I would like to know if there is something because that bothers me. There are many mosquitoes at my house. Like, intolerable.

*Interviewer: Hmm.’*

P03, Female, entrepreneur (IDI).

Insufficient information from the health system and media channels or misinformation about the control measures were concepts that negatively influenced some individuals’ perceived self-efficacy. In addition, the lack of success and not experiencing beneficial results after implementing mosquito control measures also was another concept that reduced self-efficacy.

‘Female L: But they say it does not matter if you cover the water tanks adequately. Thus the mosquitoes will find a way to enter (reach the water).

Interviewer: So you heard this?

*Female L: Hmm, yes.*

Interviewer: Where did you hear this?

*Female L: From people.*

Interviewer: Just from people.’

P06, Female, teacher (IDI).

‘Male L: You cannot avoid them, some breeding sites, but the mosquitoes will further develop, although you are paying attention.

Interviewer: Thus, you have the perception that it is impossible to destroy all breeding sites.

Male L: I believe, It is difficult, or you need to find a way, use a magnifying glass in the gardens. However, you do not have the means to do so.

*Interviewer: To do so.*

Male L: Thus, in general, to combat mosquitoes, I believe it is something very complex.’

*P19, Male, teacher (IDI).*

As portrayed by the quotes, insufficient knowledge and misinformation about control measures and the perception that it is impossible to control mosquitoes reduced some participants’ self-efficacy.

### Internal and external locus of control

All participants had a sense of individual and collective responsibility for controlling mosquitoes. The participants were asked who is responsible for preventing and controlling ABIDs; they explained that the individual, the community, and the government, including the health system, are responsible for preventing and controlling the mentioned diseases. All the interviewed participants felt they were accountable for their well-being (internal locus of control). Still, they indicated that the health system was also responsible for preventing and controlling ABIDs during epidemics. The health system needs to make the regulations, develop interventions and support the community with the control of mosquitoes. Table 3 shows opinions on the above topic from the point of view of three generations living on the island.

**Table 3:** Opinions of three generations concerning prevention and control of ABIDs

Generations	Opinions
Youth (high school students)	*Boy 1: It is not necessarily what you (the community and the health system) are doing. It depends on which stage of the process you are in. Are you (the health system and the community) trying to solve the problem by preventing mosquitoes from biting people? It should prevent the mosquitoes from being in the area in the first place* *Moderator: Okay* *Boy 1: That is why you have things like preventing standing water, cleaning the trash, removing mosquito breeding sites, and things like that* *Moderator: Do you (all students) think that the government needs to be more involved in, for example, cleaning the island?* *Boy 2: Yes* *Girl 1: Yeah* *Boy 2: Yeah, not just for mosquitoes, just because we need to be trash less (less illegal dumping sites and waste that cannot be recycled)* *Girl 1: Yeah* *FGD with high school students of the international school*
Adult	*Female G: I believe that we (all) are responsible* *Interviewer: Yes?* *Female G: I believe we (all) are responsible; if you start at your house, if each person starts at their house, I think we are a step ahead* *Interviewer: Okay* *Female G: If the government provides its tools to support. If the government puts containers in neighbourhoods, people can dispose of their waste* P08, Female, inspectress, 54 years old (IDI)
Elderly	*Interviewer: When prevention and control actions need to be implemented for these diseases, who is responsible?* *Female G: Everybody* *Interviewer: Everybody* *Female G: Each on its own* *Interviewer: With that, you mean the community or the government?* *Female G: The community and the government because the government is part of the community* P11, Female, retired, 81 years old (IDI)

When governmental involvement is lacking, participants indicated that they do not feel motivated to participate in mosquito control. Collective responsibility plays an essential role in the interviewed participants’ decision-making and preventive behaviour.

After examining the empirical material through different concepts of the HBM, we have learned that most participants performed preventive behaviour to protect themselves against ABIDs or were aware of the actions that could be done to prevent ABIDs. We found that preventive behaviours were activated when people had a high-risk perception (high perceived susceptibility and severity of disease), which was motivated by increased internal and external cues to action. The participants from the FGDs and IDIs indicated that they used repellents, Abate, larvivorous fish, and screens for doors and windows to protect themselves against mosquitoes. Also, removing mosquito breeding sites was often mentioned as a control action when they felt at risk of acquiring ABIDs. HSB was shaped not only by the concepts of the HBM but also by the contexts in which mosquito control occurs. The distrust in the government, including the health system, the lack of community participation in mosquito control and the unsolved issues with illegal dumping sites discouraged individuals from participating in the prevention and control of ABIDs.

## Discussion

This study aimed to understand the intricate processes influencing individuals’ decision-making and HSB concerning the prevention and control of ABIDs. In this study, both internal and external cues to action indirectly influenced HSB through the impact of perceived threats (perceived susceptibility and severity). Low media coverage and internal cues to action (limited or no experience with symptoms of a disease) were linked with a low perceived threat. This result is in agreement with the findings of two other studies (among different study populations, including people of all SES and age categories) performed in Curaçao [18, 19]. Subsequently, we found that measures to control mosquitoes were not performed when the perceived threat of disease was low. Another study that researched the association between cues to action, perceived threat, and HSB indicated that internal and external cues arose an individual’s perceived threat by influencing the perceived susceptibility and severity of a disease, which led to the performance of preventive behaviours [24].

Most participants (in all age categories) in our study showed good knowledge about the prevention and control measures of ABIDs. However, we found that good knowledge did not guarantee the performance of ABIDs control actions or the translation of knowledge into practice. A study in Venezuela indicated that despite a high knowledge level concerning measures to avoid mosquito bites, potential mosquito breeding sites were still present in two-thirds of the examined properties [25]. In our study, properties were not examined to verify the mentioned results. However, most participants reported that they did not always protect themselves against mosquito bites and did not remove mosquito breeding sites frequently, even though they knew those actions were required to prevent ABIDs.

In this study, the perceived barriers played a prominent role in the decision-making process and HSB of the participants. The perceived barriers prevail over the benefits of preventive and control interventions to reduce the risk of ABIDs. Another study conducted in Northeast Thailand also found that the barriers to dengue control overshadowed the perceived benefits [26]. In our study, the following barriers influenced HSB negatively: (i) the perceived passive attitude of the health system, including the government, (ii) lack of community involvement, (iii) the perception that mosquito control is challenging, and (iv) issues with waste management. Our previously published articles have also addressed the mentioned barriers [16, 18, 19, 27].

The participants reported that they were/are responsible for protecting themself from ABIDs (internal locus of control). However, they also indicated that if the health system and the community are not participating in mosquito control, they have a higher risk of acquiring ABIDs. During the last epidemics of ABIDs and at the time of data collection, the participants believed that the health system and the community had a passive attitude toward mosquito control. In other words, there is no collective responsibility for the implementation of mosquito control interventions. We found that the mentioned perception obstructed community engagement and participation in mosquito control in Curaçao.

Furthermore, the mosquitoes have more breeding sites due to the illegal dumping sites scattered throughout the island, increasing the mosquito abundance and risk for disease transmission [27]. The community is aware of the negative consequences of the illegal dumping sites and perceives their efforts as ineffective. More efforts are needed to build trust and close the gaps between the community and the health system to improve community participation in mosquito control. The World Health Organization suggests that community involvement and engagement are essential for effective and sustainable results [28]. Mosquito control without community participation is not sustainable. A recently published systematic review and meta-analysis covering Latin America and Caribbean studies found statistically significant and relevant public health outcomes in pooled effectiveness estimates for interventions focusing on health education and community engagement [12].

Based on our data, the following recommendations can be provided to work towards a more bottom-up approach. The health system needs to lead by example since the community needs a role model in mosquito control. The health system needs to develop mosquito control and waste management regulations. Regulations and enforcement concerning illegal dumping are required to support mosquito control interventions within the community. The community agreed that enforcement with consequences for the lawbreakers is essential to maintain respect for the regulations. Key decision-makers need to forge partnerships with community leaders for better communication and collaboration with the community. The participation of community leaders can stimulate social pressure among community members. Community participation can also be stimulated by sending reminders about mosquito control via traditional (e.g., television, radio, newspapers) and modern (e.g., social media or SMS) communication channels. SMS is a good option since not everyone has internet. Our participants indicated that they prefer visual communication over written communication. Furthermore, an educational curriculum needs to be developed to motivate and educate the younger generation; they are the future adults who eventually need to perform mosquito control interventions.

This study was limited by its design; since the research is based on qualitative methods, the findings cannot prove causality. Although proving causality is essential in science, this study aimed to understand decision-making processes and HSB regarding preventing and controlling ABIDs among individuals living in Curaçao. For this type of study, qualitative methods are required. Qualitative studies provide an explicit rendering of the structure, order, and patterns found among a group. To increase the credibility of the findings, two types of data collection methods were used to validate the information collected. These two methods help produce a more comprehensive set of findings to better understand the intricate processes underlying individuals’ decision-making and HSB.

## Conclusion

This multi-method qualitative study used the HBM to understand concepts that influenced decision-making and the HSB of individuals. We found that cue to action played an important role in attenuating and amplifying risk perception concerning ABIDs. Our findings shed light on the view that having good knowledge of preventing ABIDs is insufficient to activate HSB. A critical barrier that needs to be addressed is the health system's and community's passive attitude toward mosquito control. The collective responsibility and collaboration between the community and the health system seem to be the key to improving mosquito control interventions. Efforts are needed to make the health system mosquito control interventions visible, and interventions need to be developed to increase trust and stimulate community participation. Our findings and recommendations can be used in health policies and interventions to improve community participation in *Aedes* control in Curaçao.

### Supplementary Information

**Additional file 1.** **Text S1. **Topic guide FGD

**Additional file 2.** **Text S2. **Topic guide IDI

**Additional file 3.** **Table S1. **Coding List

## Data Availability

The data supporting this article’s conclusions and recommendations are included in the article. The raw data will not be publicly available because participants did not consent to have their full transcript available. Requests to access the data can be sent to Ieneke van der Gun, e-mail address: b.t.f.van.der.gun01@umcg.nl, and Eurydice Martina (project coordinator at Curaçao Biomedical & Health Research Institute), e-mail address: e.martina@cbhri.com.

## Abbreviations

ABIDs: *Aedes*-Borne infectious diseases
F: Female
FGDs: Focus Group Discussions
HBM: Health Belief Model
HSB: Health-seeking Behaviour
IDIs: In-dept Interviews
M: Male
SES: Socioeconomic Status
