# Supplementary material for: Contexts motivating protective behaviours related to Aedes-borne infectious diseases in Curaçao
Source: BMC Public Health. 2023 Sep 5;23:1730. doi: 10.1186/s12889-023-16624-5 (PMC10481474; doi:10.1186/s12889-023-16624-5)
Supplement: Supplementary file 1 — Additional file 1. Text S1. Topic guide FGD [file 12889_2023_16624_MOESM1_ESM.docx]

**Text S1 Topic guide FGD**

**Group:** Community

**FGD number : ……………………………… Moderator : ………………………………**

**Date : ……………………………… Note-taker : ………………………………**

**Introduce yourself to the participants:**

Thank you very much for accepting to participate in this group discussion. My name is Vaitiare Jansen. I am a doctoral student at the University of Groningen.

- ***Explain the general purpose of the study***: The general purpose of the study is to understand the performance of the health system, risk communication and behaviour of individuals from the point of view of the community in order to provide the health system with content-specific advice to strengthen risk communication efforts and sustainability of risk management.
- ***Estimated time***: Approximately 1 ½ hour
- ***Right to participate and withdraw from the study:*** Involvement in this study is voluntary. You are free to withdraw from the study at any time. You are free to skip any questions you prefer not to answer during the discussion.
- ***Use of tape recorder***: To keep a more accurate record of our discussion, I propose using a tape recorder, if you do not mind. Do you mind if I use a tape recorder? *(observe whether people agree)*
- ***Plan to protect the identity of the participants:*** The information that we will discuss here today will remain anonymous. Your names will be removed from the data, and no one will be able to link your name with what is said. No one apart from the research team will have access to the data. This data will be published and shared with the scientific community, but your name will not appear in any of the publications.
- ***Basic principles:***

1. Respecting the opinions of others is essential.
2. There are no right and wrong answers. We value each idea, opinion and experience.
3. One person speaks at a time.
4. Ask if there is any question.

- Do you have any questions?
- ***Consent:*** Do you agree to take part in this discussion?
- The moderator turns on the digital recorder and starts the discussion

**Introduction**

- As an introduction, let us go around so that you can introduce yourselves and tell us your name, age, whether you are currently working, and what type of work you do.

Let us start our discussion by talking about chikungunya and Zika. Most individuals living in Curaçao have witnessed the chikungunya infection outbreak in 2014-2015 and, more recently, the Zika infection outbreak in 2016. I heard that information about chikungunya and Zika was shared by different information channels.

1. What do people know about chikungunya?

**Probe for:**

1. Ask for the following types of information *(transmission routes and prevention measures)* if they are not mentioned.
2. Where do people obtain information about chikungunya?
3. What do people know about Zika?

**Probe for:**

1. Ask for the following types of information *(transmission routes and prevention measures)* if they are not mentioned.
2. Where do people obtain information about Zika?

**1: Risk perception**

1. What kind of people is at risk for chikungunya?

**Probe for:**

- 1. Who can be more at risk for chikungunya?
  2. Who can be less at risk for chikungunya?

1. What are the consequences of having chikungunya?

**Probe for:**

- 1. What are the social consequences?
  2. What are the economic consequences?
  3. What are the physical consequences?
  4. What are the emotional consequences?

1. What kind of people is at risk for Zika?

**Probe for:**

- 1. Who can be more at risk for Zika?
  2. Who can be less at risk for Zika?
  3. What are the consequences of having Zika?

**2: Risk communication**

1. What did you hear from the government about chikungunya and Zika?

**Probe for:**

1. Which channels were used? *(e.g. vector control inspectors, Facebook, the website of the G&Gz, folder, App etc.)*
2. What do you think about the information that you received from the government?

**Probe for:**

1. Was the information on time?
2. Was the information clear?
3. What do people around you do with the information they receive from the government?

**Probe for:**

1. Why?
2. Was it easy to accept?
3. What would be the best way to provide your community with information about chikungunya and Zika?

**Probe for:**

1. Via which channels? Why?
2. What would they like to know?
3. When do they want to receive information?

**Topic 3: Performance of the health system**

1. What did the health institution do during the chikungunya epidemic?

**Probe for:**

1. Ask for the following institutions *(G&Gz, especially for the vector control unit, hospital and health insurances)* if they are not mentioned.
2. What went well?
3. What can be improved?
4. What did the health institutions do during the Zika epidemic?

**Probe for:**

1. Ask for the following institutions *(G&Gz, especially for the vector control unit, hospital and health insurances)* if they are not mentioned.
2. What went well?
3. What can be improved?
4. How did the health professionals react during the chikungunya epidemic?

**Probe for:**

1. Ask for the following health professionals *(GP, alternative medicine practitioner, vector control inspectors)* if they are not mentioned.
2. What was their behaviour?
3. What was their reaction during the Zika epidemic?
4. What are the challenges the government faces when dealing with chikungunya and Zika?

**Probe for:**

1. Aks if the following factors *(trust issues, limited resources, immigrants, and unplanned urbanisation)* if not mentioned.
2. Why?

**Topic 4: Health care utilisation**

1. Many people suffer from chikungunya. What were the symptoms?

**Probe for:**

1. And what about Zika?
2. Which medication (s) did they use for chikungunya?

**Probe for:**

- 1. Why?
  2. Which medication did they use for Zika?
  3. Why?

1. Whom did they consult for help?

**Probe for:**

- 1. Ask for the following caregivers *(e.g. partner, parent, friend, religious leader, community worker, general practitioner or alternative medicine practitioner)* if they are not mentioned.
  2. Why? *(e.g. in the case of the GP, for sickness certification).*

1. During the chikungunya and Zika epidemic, what difficulties did people have in obtaining healthcare?

**Probe for:**

- 1. Medication costs – consultation and medicine costs
  2. Availability of medicines
  3. Waiting time before consultations
  4. Operating hours (e.g. GP availability during the evening and weekend)
  5. The attitude of health professionals
  6. Alternative medicine practitioners

**Topic 5: Health-seeking behaviour and preventive measures**

1. When prevention needs to be conducted, who all are responsible?

**Probe for:**

1. Ask for the following individuals/groups/institution *(infected individuals, not infected individuals, the community, and the government)* if they are not mentioned.
2. Why are they responsible for prevention?
3. What problems do people have in protecting themselves against chikungunya and Zika?

**Probe for:**

1. Ask for the following factor *(lack of practical information, poverty and availability of preventive tools)* if they are not mentioned.

We also heard that people do not perform preventive measures because of their mentality.

1. Could you explain what people mean by this statement?
2. What can be done to improve the community and government collaboration in the context of prevention?

**Closing question**

1. Imagine next year. We have another disease transmitted by mosquitoes. Do you think we are prepared to deal with it?

**Probe for:**

1. What can be done?

We are now reaching the end of the discussion. I want to thank you all very much for your participation in this discussion, your experiences and opinions are valuable to assist in improving risk communication and risk management in Curaçao. Does anyone have any further comments to add before we conclude this group discussion?
