# Supplementary material for: Contexts motivating protective behaviours related to Aedes-borne infectious diseases in Curaçao
Source: BMC Public Health. 2023 Sep 5;23:1730. doi: 10.1186/s12889-023-16624-5 (PMC10481474; doi:10.1186/s12889-023-16624-5)
Supplement: Supplementary file 2 — Additional file 2. Text S2. Topic guide IDI [file 12889_2023_16624_MOESM2_ESM.docx]

**Text S2 Topic guide IDI**

**IDI number: ………………………………**

**Date: ………………………………**

**Interviewer: ………………………………**

**Introduce yourself to the participants:** Thank you very much for accepting to participate in this research. My name is Vaitiare Mulderij-Jansen. I am a doctoral student at the University of Groningen.

- ***Explain the general purpose of the study***: The general purpose of the study is to understand the risk communication and behaviour of individuals concerning the prevention and control of chikungunya, dengue, and Zika, from your point of view. Your perceptions, opinions and experiences can help us provide the government with content-specific advice to strengthen risk communication efforts, the sustainability of risk management and enhance the health-seeking behaviour of people living in Curaҫao.
- ***Estimated time***: Approximately 1 hour
- ***Right to participate and withdraw from the study:*** Involvement in this study is voluntary. You are free to withdraw from the study at any time. You are free to skip any questions you prefer not to answer during the interview.
- ***Use of tape recorder***: To keep a more accurate interview record, I propose using a tape recorder, if you do not mind. Do you mind if I use a tape recorder? *(observe whether people agree)*
- ***Plan to protect the identity of the participants:*** The information that we will discuss here today will remain anonymous. Your name will be removed from the data, and no one will be able to link your name with what is said. No one apart from the research team will have access to the data. This data will be published and shared with the scientific community, but your name will not appear in any of the publications.
- ***Basic principles:***

1. There are no right and wrong answers. I value each idea, opinion and experience.
2. Ask if there is any question.

- Do you have any questions?
- ***Consent:*** Sign the “informed consent” form.
- The interviewer turns on the digital recorder and starts with the interview.

**Introduction**

- As an introduction, can you introduce yourself? Please tell me your name, age, neighbourhood, whether you are currently working, and what type of work you do.

Let us start the interview by talking about chikungunya, dengue, and Zika. Most individuals living in Curaçao witnessed the dengue outbreak in 2010, the chikungunya outbreak in 2014-2015 and the Zika outbreak in 2016.

1. What do you know about chikungunya?

**Probe for:**

1. Ask for the following types of information *(e.g. transmission routes, prevention measures, the link between these diseases, symptoms, treatment)* if they are not mentioned.
2. What do you know about dengue?

**Probe for:**

1. Ask for the following types of information *(e.g. transmission routes, prevention measures, the link between these diseases, symptoms, treatment)* if they are not mentioned.
2. What do you know about Zika?

**Probe for:**

1. Ask for the following types of information *(e.g. transmission routes, prevention measures, the link between these diseases, symptoms, treatment)* if they are not mentioned.
2. According to you, what are the reasons/causes of these outbreaks in Curaçao?

**Probe for:**

1. What makes Curaçao susceptible to these diseases?
2. How did you obtain or receive information about these diseases?

***Infodemic:*** *Link inaccurate information with the source of information.*

**1: Risk perception**

1. Who are at risk for chikungunya?

**Probe for:**

- 1. Who can be more at risk for chikungunya? *Ask for waste management officials or employees of the VCU if they are not mentioned.* Why?
  2. Who can be less at risk for chikungunya? Why?
  3. And in the case of dengue? Why?
  4. And in the case of Zika? Why?

1. What kind of people do you think are more at risk for complications in the case of chikungunya?

Probe for:

- 1. Why?
  2. And in the case of dengue? Why?
  3. And in the case of Zika? Why?

1. Do you consider yourself at risk for one of these diseases, and why or why not?
2. Which diseases do you consider more severe or threatening to your health?

**Probe for:**

- 1. Explain why or why not.

1. What can influence your feeling of being at risk?

**Probe for:**

- 1. Time? Is there a change over time?
  2. Does media attention has an influence?
  3. Does the recovery rate have an influence?

1. Which impact did the community, family, or friend have on your feeling of being at risk?

**Probe for:**

1. In what way or not?
2. Has this impact affected your behaviour in some way?
3. What were/are the personal consequences of you having chikungunya? Or What can be the personal consequences of having chikungunya?

**Probe for:**

- 1. What are the social, economic, physical and psychological consequences?
  2. What can be the consequences for the family of those who had chikungunya?

1. What were/are the personal consequences of your having Zika? Or What can be the personal consequences of having Zika?

**Probe for:**

- 1. What are the social consequences, economic, physical, and psychological consequences?
  2. What can be the consequences for the family of those who had Zika?

1. What were/are the personal consequences of your having dengue? Or What can be the personal consequences of having dengue?

**Probe for:**

- 1. What are the social, economic, physical, and psychological consequences?
  2. What can be the consequences for the family of those who had dengue?

1. Which of these consequences affected the choices you made or the actions you took to protect yourself during the outbreaks?

**Probe for:**

- 1. Which actions did you perform *(e.g. personal and household protection and mosquito breeding sites control)*?
  2. Why?
  3. Do these consequences still affect your present choices or actions for protection?
  4. Furthermore, what do you do currently for protection and prevention?

**2: Risk communication**

**Definition of risk communication:** *Risk communication refers to the exchange of real-time information, including advice and opinions between experts and individuals facing threats to their health, economic or social well-being.*

1. What did you hear from the government (official information) about chikungunya?

**Probe for:**

1. What about Zika?
2. What about dengue?
3. Which channels were used? *(e.g. vector control inspectors, Facebook, the website of the G&Gz, folder, App etc.)*
4. Which impact did the official information have on you?

**Probe for:**

1. In what way or not?
2. What did you do with the received official information?
3. Did you use it to educate others, and why not?
4. What do you think about the information that you received from the government?

**Probe for:**

1. Was the information on time, clear, and useful?
2. Why or why not?
3. Do you trust the information that the government provides? Explain why or why not.

**Probe for:**

1. In the case of distrust, how do you deal with this?
2. Which sources other than the government do you trust?
3. How do you ensure the authenticity of the information?
4. After the outbreaks, did you receive or continue to receive official information to prevent these outbreaks in the future?

**Probe for:**

1. According to you, is this positive or negative? Why or why not?
2. Does this influence your (preventive) behaviour?
3. According to you, what are the factors that obstruct the risk communication of the government concerning these diseases?
4. What can be done to improve risk communication?

**Probe for:**

1. What would be the best way to provide you with information about these diseases?
2. Via which channels? Why?
3. What would you like to know?
4. When do you want to receive the information?

**Topic 3: Health-seeking behaviour, prevention and control measures**

**Definition of prevention:** *The action of stopping something, in this case, infection of dengue, Zika, and chikungunya from happening.*

**Definition of control measures:** *Actions and/or activities that are taken to prevent, eliminate or reduce the occurrence of infection of dengue, Zika, and chikungunya.*

1. When prevention and control measures need to be conducted, who all are responsible?

**Probe for:**

1. Ask for the following individuals/group/institution *(e.g. the participant itself, infected individuals, not infected individuals, the community, and the government)* if they are not mentioned.
2. Why or why not are they responsible for prevention and control measures?
3. Which barriers do you encounter in protecting yourself against dengue, chikungunya and Zika?

**Probe for:**

1. Ask for the following factor *(e.g. lack of practical information, financial factors, availability and accessibility of preventive tools)* if they are not mentioned.
2. Which external factors (e.g. illegal dumping sites, inadequate waste management) obstruct you in preventing and controlling these diseases?
3. In what way do they obstruct your actions?
4. According to you, what factors obstruct the government from preventing and controlling these diseases?

**Probe for:**

1. Ask for the following factors *( e.g. financing, lack of workforce, lack of educated workforce, lack of collaboration between the community and the government)* if they are not mentioned.

I also heard that people do not perform preventive and control measures to combat these diseases because of different factors, for example, mentality.

1. Could you explain what people may mean by this statement?
2. Which other factors obstruct prevention and control measures?
3. What can be done to improve the collaboration between the community and the government in preventing and controlling these diseases?

**Closing question**

1. Imagine, next year, we have another disease transmitted by mosquitoes. Do you think we are prepared to deal with it?

**Probe for:**

1. What can be done?

We are now reaching the end of the interview. I want to thank you for your participation; your experiences and opinions are valuable to assist in improving risk communication and risk management in Curaçao. Do you have any further comments to add before we conclude this interview?
