# Supplementary material for: Contexts motivating protective behaviours related to Aedes-borne infectious diseases in Curaçao
Source: BMC Public Health. 2023 Sep 5;23:1730. doi: 10.1186/s12889-023-16624-5 (PMC10481474; doi:10.1186/s12889-023-16624-5)
Supplement: Supplementary file 3 — Additional file 3. Table S1. Coding List [file 12889_2023_16624_MOESM3_ESM.docx]

**Table S1. Coding List**

| **Themes**  **(n=5)** | **Categories**  **(n=12)** | **Codes**  **(n=75)** |
| --- | --- | --- |
| Perceived threat | Perceived susceptibility Perceived severity  Knowledge about ABIDs  Cues to action  Trust | Elderly/children/women/people with chronic diseases are more at risk  Same risk for everyone  No threat to human life  No risk for men  Rainy season  Work environment  The environment around the house  Aetiology  Symptoms of Zika  Symptoms of dengue  Symptoms of Chik  Knowledge about mosquitoes  Consequences of ABIDs  Knowledge about breeding sites  Channels of information  Social pressure  Lack of information  Misinformation  Influence of religion  Internal cues to action  Health education at school  Communication between people  Risk communication of the health system  Trust in the health system  Trust in the media channels  Trust in the community  Trust in science  Trust in God |
| Barriers | Perceived barriers  Perceived benefits | Perceived benefits of actions  Lack of education regarding prevention/control  No access to the health system  Poverty  No consequences for inappropriate behaviour  Mentality of people  Time to invest in prevention/control actions  Resources  Infrastructure of neighbourhoods  Priority to prevent/control ABIDs  Connection with the environment  Mosquito control is challenging  Do not like repellents  Collaboration between the health system and community  Actions of others  Effectiveness of control measures  Negative impact of insecticide |
| Self-efficacy | Self-efficacy | People do not like to read/ educate themselves  Lack of practical information  Misinformation |
| Internal and external locus of control | Internal locus of control  External locus of control | God will protect me  The health system will protect me  I am responsible for my protection  The community is responsible for my protection  Prevention/control of ABIDs is the task of all |
| Health-seeking behaviour | HSB  Recommendations | Usage of vitamines  Usage of herbs  Usage of repellents  Screens at doors/windows  Usage of long sleeves  Usage of insecticide  Usage of larvicide  Usage of biological control measures  Removing mosquito breedings sites  Other ways to protect against mosquitoes  Reasons to not protect against mosquitoes  Social control  Interventions to clean the environment  Regulations  Research  Improve respect for authorities  Improve collaboration between the health system and the community  Recommendations to motivate HSB  Health education at schools  Consequences for wrongdoing  Recommendations to improve risk communication  Improve trust in the health system |
